# Supplementary material for: Semi-Synthetic Dihydrotestosterone Derivatives Modulate Inherent Multidrug Resistance and Sensitize Colon Cancer Cells to Chemotherapy
Source: Pharmaceutics. 2023 Feb 9;15(2):584. doi: 10.3390/pharmaceutics15020584 (PMC9966060; doi:10.3390/pharmaceutics15020584)
Supplement: Supplementary file 1 [file pharmaceutics-15-00584-s001.zip › pharmaceutics-2115563-supplementary.pdf]

pharmaceutics

Article

Semi-Synthetic Dihydrotestosterone Derivatives Modulate Inherent Multidrug Resistance and Sensitize Colon Cancer Cells to Chemotherapy

Ferenc István Nagy [1], Dóra Izabella Adamecz [1], Ádám Baji [2], Ágnes Kiricsi [3], Ildikó Huliák [1], Andrea Rónavári [4], Zoltán Kónya [4], Éva Frank [2], Mohana Krishna Gopisetty [1,5,*] and Mónika Kiricsi [1,*]

1 Department of Biochemistry and Molecular Biology, Doctoral School of Biology, University of Szeged, Közép Fasor 52, H-6726 Szeged, Hungary
2 Department of Organic Chemistry, University of Szeged, Dóm tér 8, H-6720 Szeged, Hungary
3 Department of Oto-Rhino-Laryngology, Head and Neck Surgery, University of Szeged, Tisza Lajos krt. 111, H-6720 Szeged, Hungary
4 Department of Applied and Environmental Chemistry, University of Szeged, Rerrich tér 1, H-6720 Szeged, Hungary
5 Molecular Genetics, German Cancer Research Center (DKFZ), Im Neuenheimer Feld 280, 69120 Heidelberg, Germany
* Correspondence: mohanakrishna.gopisetty@dkfz-heidelberg.de (M.K.G.); kiricsim@bio.u-szeged.hu (M.K.); Tel.: +36-62-544887 (M.K.)

Contents:

Table S1: Data of IC_{50} determination for chemotherapy drugs
Table S2: MTT combinational treatments
Table S3: Sequence and final concentrations of primers used in the study
Table S4: Data of RT-qPCR measurements of ABC transporter expression
Table S5: Data of densitometric analysis of ABCB1 expression
Table S6: Data of RT-qPCR measurements of endoplasmic reticulum stress
Table S7: Data of densitometric analysis of BIP
Table S8: Data of viability of Colo 320 and Colo 205 cells after steroid treatment

Table S1: Data of IC_{50} determination for chemotherapy drugs

Bleomycin		Carmustine		Cisplatin		Doxorubicin		Epirubicin	
C [µM]	Mean ± SD	C [µM]	Mean ± SD	C [µM]	Mean ± SD	C [µM]	Mean ± SD	C [µM]	Mean ± SD
Control	100.0 ± 6.1	Control	100.0 ± 1.3	Control	100.0 ± 12.9	Control	100.0 ± 8.5	Control	100.0 ± 0.2
4.14	99.3 ± 8.5	608	69.7 ± 7.9	1.62	64.9 ± 3.5	115	80.2 ± 0.6	14.37	74.1 ± 5.4
8.28	97.5 ± 16.2	1217	34.7 ± 3.5	3.24	65.1 ± 12.4	460	30.3 ± 6.6	28.75	82.2 ± 8.8
16.6	102.1 ± 11.6	2433	8.8 ± 1.5	6.49	61.6 ± 3.4	920	18.7 ± 6.4	57.50	52.8 ± 4.9
33.1	89.4 ± 15.5	4866	5.7 ± 0.6	13.0	62.1 ± 14.2	3680	11.6 ± 1.2	115.0	17.6 ± 2.9
132	61.1 ± 20.2	9733	5.5 ± 0.8	25.9	44.1 ± 7.1			230.0	13.7 ± 0.4
265	44.9 ± 18.0	19466	4.2 ± 0.3	51.9	22.8 ± 2.5			460.0	16.1 ± 0.4
530	32.8 ± 12.0	38932	4.1 ± 0.1	104	21.2 ± 2.0			920	15.9 ± 2.7

Table S2: MTT combinational treatments. Table representing the results of the viability screens on Colo 320 cells treated with various combinations of chemotherapeutic drugs (at the indicated concentrations) and androstano-arylpyrimidines. The effective combinations at the lowest drug concentrations are highlighted with green colorThe viability of the cells receiving combinational treatments were compared to those subjected to the chemotherapy drug alone. Fisher's LSD test, *: P < 0.05, **: P < 0.01, ***: P < 0.001, ****:P < 0.0001 ns: non-significant.

**Drugs**	**Drug concentrations**	**Androstano-arylpyrimidines**				
		20 µM				
	µM	**10a**	**10d**	**10e**	**10f**	**10g**
**Bleomycin**	45	ns	ns	ns	ns	****
	68	ns	ns	ns	ns	****
	98	ns	ns	ns	ns	****
	143	ns	ns	ns	ns	****
	215	ns	ns	ns	****	****
**Carmustine**	491	**	***	**	ns	ns
	603	*	****	****	**	ns
	729	**	ns	**	ns	ns
	881	***	ns	*	**	ns
	1083	**	ns	***	*	ns
**Cisplatin**	2	ns	ns	ns	ns	ns
	4	ns	ns	ns	**	*
	8	ns	ns	ns	ns	****
	16	ns	ns	ns	ns	ns
	36	ns	ns	ns	ns	ns
**Doxorubicin**	112	ns	ns	ns	ns	***
	149	ns	ns	ns	ns	**
	194	ns	ns	ns	ns	ns
	252	ns	**	ns	ns	**
	336	ns	ns	ns	ns	ns
**Epirubicin**	19	****	****	****	****	ns
	27	****	****	****	****	ns
	37	ns	ns	ns	*	ns
	50	ns	ns	ns	*	ns
	69	ns	ns	ns	ns	ns

Table S3: Sequence and final concentrations of primers used in the study

**Gene**	**Forward primer**<br>**Reverse primer**	**Final concentration [nM]**
GAPDH	5'-TGCACCACCAACTGCTTAGC-3'<br>5'-GGCATGGACTGTGGTCATGAG-3'	200
ABCB1	5'-GAGCCTACTTGGTGGCACAT-3'<br>5'-TCCTTCCAATGTGTTCGGCA-3'	200
ABCC1	5'-CATGCTCACTTTCTGGCTGGT-3'<br>5'-GTCCACCTGGGCATCCTCTTTT-3'	400
ABCC4	5'-CGTGTTCTTCTGGTGGCTCA-3'<br>5'-TCCCAGAACCCTTGCAACTC-3'	200
ABCC5	5'-ATGCTCGGGAGCGTGGTTG-3'<br>5'-TTTCACATCAGAATTCCTGCGCC-3'	400
ABCC10	5'-CTCTCCTCACAGATGCCAAGAT-3'<br>5'-AGGATCGTGTTGAGCCTATGG-3'	400
BIP	5'-TGTTCAACCAATTATCAGCAAACTC-3'<br>5'-TTCTGCTGTATCCTCTTCACCAGT-3'	200
CHOP	5'-GGAGCATCAGTCCCCCACTT-3'<br>5'-TGTGGGATTGAGGGTCACATC-3'	200
ATF4	5'-GGCCACCATGGCGTATTA-3'<br>5'-TGCTGAATGCCGTGAGAA-3'	200
ATF6	5'-CTTTTAGCCCGGGACTCTTT-3'<br>5'-TCAGCAAAGAGAGCAGAATCC-3'	200
GRP94	5'-CAGTTTTGGATCTTGCTGT-3'<br>5'-CAGCTGTAGATTCCTTTGC-3'	200
XBP1s	5'-AACCAGGAGTTAAGACAGCGCTT-3'<br>5'-CTGCACCCTCTGCGGACT-3'	200
XBP1t	5'-CTGAATCTGAAGAGTCAATACCGCCAGAAT-3'<br>5'-AGGAGTTAAGACAGCGCTTGGGGATGGAT-3'	200
NF-KB	5'-AGCACAGATACCACCAAGACCC-3'<br>5'-CCAGGGAGATGCGCACTG-3'	200

Table S4: Data of RT-qPCR measurements of ABC transporter expression

Mean ± SD	ABCB1	ABCC1	ABCC4	ABCC5	ABCC10
10a	2.72 ± 0.01	1.31 ± 0.17	1.41 ± 0.22	2.43 ± 0.12	1.87 ± 0.21
10d	1.12 ± 0.01	0.92 ± 0.05	1.27 ± 0.17	1.36 ± 0.12	1.24 ± 0.02
10e	0.62 ± 0.01	1.08 ± 0.15	0.94 ± 0.10	0.93 ± 0.15	0.77 ± 0.09
10f	1.52 ± 0.03	1.35 ± 0.11	1.08 ± 0.11	1.65 ± 0.15	1.22 ± 0.07
10g	1.43 ± 0.08	2.00 ± 0.23	1.41 ± 0.20	1.93 ± 0.13	1.32 ± 0.05

Table S5: Data of densitometric analysis of ABCB1 expression

	Control	Tunicamycin	10a	10d	10e	10f	10g
Mean ± SD	93182 ± 16560	132439 ± 37564	117779 ± 2819	116783 ± 14870	83740 ± 14555	117388 ± 20348	117034 ± 3961

Table S6: Data of RT-qPCR measurements of endoplasmic reticulum stress

Mean ± SD	ATF4	ATF6	BIP	CHOP	EDEM	GRP94	NFKB	XBP1t	XBP1s
Control	1.00 ± 0.04	1.00 ± 0.05	1.00 ± 0.02	1.00 ± 0.05	1.00 ± 0.06	1.00 ± 0.01	1.00 ± 0.00	1.00 ± 0.08	1.00 ± 0.07
Tuni-camycin	2.30 ± 0.04	1.19 ± 0.09	2.69 ± 0.10	4.81 ± 0.39	1.70 ± 0.19	5.35 ± 0.37	1.47 ± 0.04	2.63 ± 0.08	0.78 ± 0.25
10a	2.15 ± 0.20	2.15 ± 0.19	6.18 ± 0.17	14.33 ± 1.01	1.76 ± 0.29	7.03 ± 0.4	1.35 ± 0.03	4.71 ± 0.31	6.54 ± 2.15
10d	1.46 ± 0.12	1.33 ± 0.18	2.27 ± 0.13	2.02 ± 0.09	1.70 ± 0.16	1.26 ± 0.34	1.48 ± 0.01	1.73 ± 0.10	2.33 ± 0.43
10e	4.18 ± 0.06	2.40 ± 0.06	2.20 ± 0.21	6.97 ± 0.61	3.42 ± 0.07	1.81 ± 0.14	2.51 ± 0.02	2.82 ± 0.1	4.85 ± 0.28
10f	1.88 ± 0.16	1.72 ± 0.08	2.49 ± 0.25	4.60 ± 0.19	1.68 ± 0.05	3.05 ± 0.17	1.72 ± 0.07	2.73 ± 0.07	3.39 ± 0.33
10g	1.53 ± 0.17	1.27 ± 0.10	1.87 ± 0.02	1.05 ± 0.10	1.64 ± 0.10	0.65 ± 0.05	1.61 ± 0.10	1.20 ± 0.03	1.67 ± 0.25

Table S7: Data of densitometric analysis of Bip

	Control	Tunicamy-cin	10a	10d	10e	10f	10g
Mean ± SD	270 ± 6.4	448 ± 24.7	472 ± 7.78	596 ± 107	589 ± 24.0	442 ± 64.3	413 ± 21.2

Table S8: Data of viability of Colo 320 and Colo 205 cells after steroid treatment

	Control	10a	10d	10e	10f	10g
Colo 320	100.0 ± 10.29	86.25 ± 5.74	85.64 ± 9. 25	86.12 ± 3.74	86.78 ± 3.74	74.73 ± 3.43
Colo 205	100.0 ± 2.0	98. 6 ± 2.5	99.5 ± 3.20	101.0 ± 4.3	94 ± 1.3	82.0 ± 3.1
